# Supplementary material for: Study of element concentrations in blood serum of patients receiving parenteral nutrition using total reflection X-ray fluorescence analysis
Source: PLoS One. 2026 Feb 2;21(2):e0341090. doi: 10.1371/journal.pone.0341090 (PMC12863542; doi:10.1371/journal.pone.0341090)
Supplement: S1 File — (PDF) [file pone.0341090.s001.pdf]

Study of element concentrations in blood serum of patients receiving parenteral nutrition using total reflection X-ray fluorescence analysis

Monika Pierzak-Stępień[1], Aldona Kubala-Kukuś[2,3], Dariusz Banaś[2,3], Ilona Stabrawa[2,3], Jolanta Wudarczyk-Moćko[3], Karol Szary[2,3], Monika Biernacka[2], Milena Piotrowska[2], Dariusz Pasieka[2], Natalia Wojtaś[2], Andrzej Dąbrowski[2,3], Stanisław Głuszek[3,4,5]

1 Institute of Health of Sciences, Faculty of Health of Sciences, Jan Kochanowski University, Kielce, Poland

2 Institute of Physics, Faculty of Natural Sciences, Jan Kochanowski University, Kielce, Poland

3 Holycross Cancer Center, Kielce, Poland

4 Hospital of the Ministry of Interior and Administration, Kielce, Poland

5 Institute of Genetics and Animal Biotechnology, Polish Academy of Sciences, Magdalenka, Poland

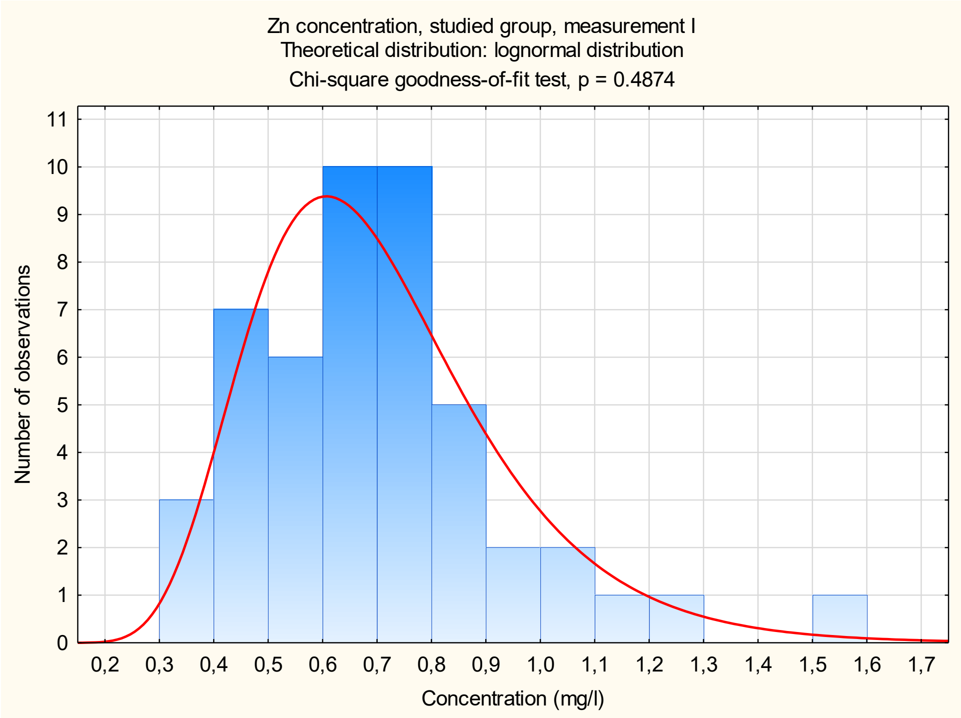

1S Fig. Distribution of Zn concentration in the serum of the patients from the studied group (measurement I). Red solid line represents lognormal distribution.

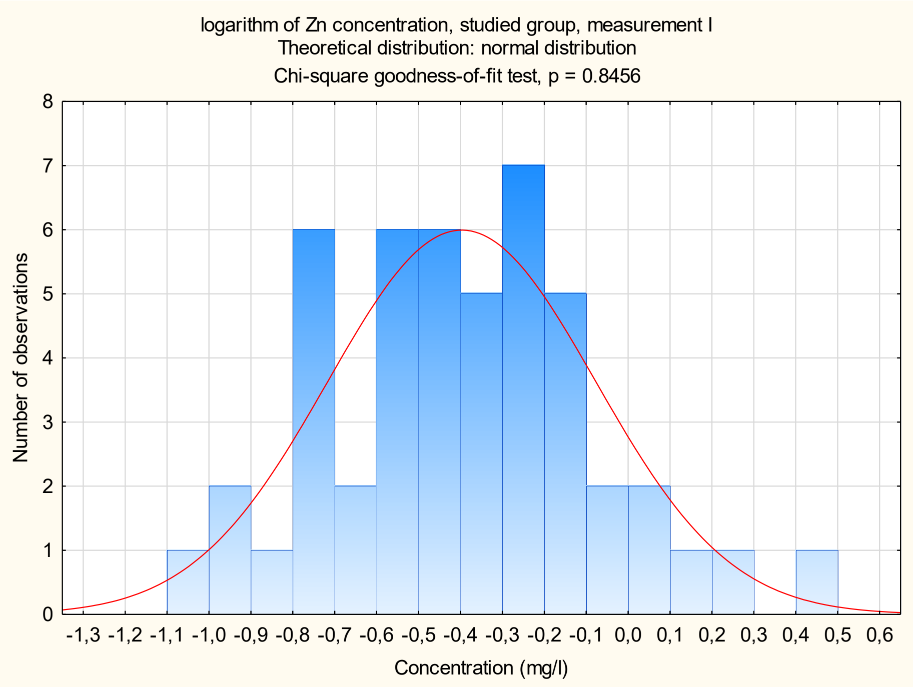

2S Fig. Distribution of logarithm of Zn concentration in the serum of the patients from the studied group (measurement I). Red solid line represents normal distribution.

1S Table. Test probability p-values for the χ^2 goodness-of-fit test for comparing experimental distributions of element contents to the theoretical lognormal distribution for the studied group (measurement I and measurement II) and for the control group.

Element	p-value	Theoretical distribution
Studied group		
Measurement I - P	p = 0.3208	lognormal
Measurement I - S	p = 0.3560	lognormal
Measurement I - Cl	p = 0.3993	lognormal
Measurement I - K	p = 0.0622	lognormal
Measurement I - Ca	p = 0.2744	lognormal
Measurement I - Cr	p = 0.3694	lognormal
Measurement I - Fe	p = 0.2200	lognormal
Measurement I - Cu	p = 0.5042	lognormal
Measurement I - Zn	p = 0.4874	lognormal
Measurement I - Se	p = 0.1571	lognormal
Measurement I - Br	p = 0.4135	lognormal
Measurement II - P	p = 0.8254	lognormal
Measurement II - S	p = 0.3336	lognormal
Measurement II - Cl	p = 0.3872	lognormal
Measurement II - K	p = 0.5121	lognormal
Measurement II - Ca	p = 0.1475	lognormal
Measurement II - Cr	p = 0.3310	lognormal
Measurement II - Fe	p = 0.1398	lognormal
Measurement II - Cu	p = 0.1035	lognormal
Measurement II - Zn	p = 0.4599	lognormal
Measurement II - Se	p = 0.1412	lognormal
Measurement II - Br	p = 0.3176	lognormal
Control group		
P	p = 0.7938	lognormal

S	p = 0.0694	lognormal
Cl	p = 0.1793	lognormal
K	p = 0.4071	lognormal
Ca	p = 0.0663	lognormal
Cr	p = 0.6008	lognormal
Fe	p = 0.1239	lognormal
Cu	p = 0.4766	lognormal
Zn	p = 0.0926	lognormal
Se	p = 0.3213	lognormal
Br	p = 0.4720	lognormal

2S Table. Comparison of mean value of element concentrations for women and men in the control group using t-test for the independent groups.

Element	Mean value (women)	Mean value (men)	p value for mean value	Number of women	Number of men	Standard deviation (women)	Standard deviation (men)	p value for variance
ln P	3.62	3.55	0.5133	35	15	0.33	0.39	0.3834
ln S	6.26	6.38	**0.0449**	35	15	0.21	0.14	0.0920
ln Cl	8.07	8.05	0.3156	35	15	0.06	0.06	0.9884
ln K	4.89	4.89	0.8685	35	15	0.13	0.10	0.4704
ln Ca	4.33	4.37	0.2438	35	15	0.12	0.09	0.2028
ln Cr	-2.97	-3.33	0.0554	35	15	0.60	0.56	0.8201
ln Fe	-0.017	0.128	0.4100	35	15	0.506	0.689	0.1392
ln Cu	0.110	0.022	0.1862	35	15	0.218	0.199	0.7399
ln Zn	-0.262	-0.189	0.3388	35	15	0.260	0.200	0.2911
ln Se	-2.94	-2.88	0.4836	35	15	0.29	0.21	0.2102
ln Br	0.818	0.755	0.4268	35	15	0.275	0.194	0.1621

3S Table. Comparison of mean value of element concentrations for women and men in the studied group (measurement I) using the t-test for the independent groups.

Element	Mean value (men)	Mean value (women)	p value for mean value	Number of men	Number of women	Standard deviation (men)	Standard deviation (women)	p value for variance
Measurement I - ln P	3.78	3.87	0.4922	27	21	0.45	0.39	0.4946
Measurement I - ln S	6.09	5.91	0.0731	27	21	0.22	0.45	**0.0005**
Measurement I - ln Cl	8.08	8.07	0.4736	27	21	0.07	0.08	0.5948
Measurement I - ln K	4.87	4.81	0.2491	27	21	0.19	0.18	0.8071
Measurement I - ln Ca	4.29	4.25	0.3628	27	21	0.14	0.09	0.0550
Measurement I - ln Cr	-3.01	-2.98	0.8608	27	21	0.43	0.62	0.0733
Measurement I - ln Fe	-0.430	-0.306	0.5555	27	21	0.722	0.714	0.9696
Measurement I - ln Cu	0.172	0.123	0.4851	27	21	0.235	0.244	0.8456
Measurement I - ln Zn	-0.322	-0.493	0.0655	27	21	0.342	0.265	0.2465
Measurement I - ln Se	-3.26	-3.12	0.2648	27	21	0.471	0.346	0.1590
Measurement I - ln Br	-0.017	0.051	0.6776	27	21	0.558	0.564	0.9494

4S Table. Comparison of mean value of element concentrations for women and men in the studied group (measurement II) using the t-test for the independent groups.

Element	Mean value (men)	Mean value (women)	p value for mean value	Number of men	Number of women	Standard deviation (men)	Standard deviation (women)	p value for variance
Measurement II - ln P	3.88	3.99	0.1917	27	21	0.21	0.37	**0.0066**
Measurement II - ln S	6.11	5.98	0.1982	27	21	0.19	0.47	**0.0000**
Measurement II - ln Cl	8.09	8.06	0.1002	27	21	0.09	0.06	0.0607
Measurement II - ln K	4.93	4.88	0.2928	27	21	0.16	0.15	0.8121
Measurement II - ln Ca	4.32	4.28	0.2797	27	21	0.13	0.11	0.3009
Measurement II - ln Cr	-3.19	-3.07	0.4472	27	21	0.57	0.54	0.7811
Measurement II - ln Fe	-0.153	0.022	0.5488	27	21	1.14	0.768	0.0760
Measurement II - ln Cu	0.265	0.276	0.8601	27	21	0.211	0.210	0.9926
Measurement II - ln Zn	-0.295	-0.352	0.5153	27	21	0.329	0.249	0.2035
Measurement II - ln Se	-3.17	-2.89	**0.0177**	27	21	0.439	0.294	0.0690
Measurement II - ln Br	0.043	0.080	0.7930	27	21	0.444	0.519	0.4482

5S Table. Result of U Mann-Whitney test for comparison of concentration distribution of S (measurement I), P (measurement II) and S (measurement I) between women and men.

Element	Sum of ranks (men)	Sum of ranks (women)	Number of men	Number of women	p value
measurement I - S	727	449	27	21	0.1767
measurement II - P	583	593	27	21	0.1050
measurement II - S	703	473	27	21	0.3942

6S Table. The probability p values for the t-test comparing the mean values of element concentrations in the studied group (I measurement and II measurement) and in the control group.

Element	studied group (measurement I) and control group		studied group (measurement II) and control group	
	p value for mean value	p value for variance	p value for mean value	p value for variance
ln P	**0.0063**	0.1510	**0.0000**	0.2351
ln S	**0.0000**	**0.0002**	**0.0000**	**0.0003**
ln Cl	0.4563	0.1227	0.4416	0.0660
ln K	0.1854	**0.0023**	0.4369	**0.0405**
ln Ca	**0.0045**	0.5343	0.0701	0.4858
ln Cr	0.4558	0.2535	0.6577	0.5260
ln Fe	**0.0025**	0.1045	0.5243	**0.0002**
ln Cu	0.1459	0.4814	**0.0000**	0.8452
ln Zn	**0.0073**	0.0631	0.1455	0.1913
ln Se	**0.0002**	**0.0025**	0.0830	**0.0070**
ln Br	**0.0000**	**0.0000**	**0.0000**	**0.0000**

7S Table. Probability p values for the nonparametric Mann-Whitney test comparing element concentration distributions in the studied group (I measurement and II measurement) and in the control group.

Element	studied group (measurement I) and control group	studied group (measurement II) and control group
	p value	p value
S	**0.0000**	**0.0000**
K	0.4491	0.2511
Fe	**0.0006**	0.1090
Se	**0.0011**	0.2084
Br	**0.0000**	**0.0000**

8S Table. The probability p values for the t-test comparing the mean values of element concentrations in the studied group of women (measurement I and II) and in the control group.

Element	studied group of women (measurement I) and control group		studied group of women (measurement II) and control group	
	p value for mean value	p value for variance	p value for mean value	p value for variance
ln P	**0.0144**	0.3540	**0.0003**	0.5368
ln S	**0.0003**	**0.0001**	**0.0033**	**0.0001**
ln Cl	0.8200	0.1421	0.3386	0.9063
ln K	0.0702	0.0670	0.9199	0.2769
ln Ca	**0.0171**	0.2205	0.0986	0.6063
ln Cr	0.9667	0.8508	0.5760	0.5957
ln Fe	0.0820	0.0745	0.8217	**0.0309**
ln Cu	0.8365	0.5532	**0.0072**	0.8767
ln Zn	**0.0023**	0.8918	0.2077	0.8505
ln Se	**0.0443**	0.3881	0.5334	0.9686
ln Br	**0.0000**	**0.0002**	**0.0000**	**0.0011**

9S Table. The probability p values for the nonparametric Mann-Whitney test comparing the element concentration distributions in the studied group of women (measurement I and II) and in the control group.

Element	studied group of women (measurement I) and control group	studied group of women (measurement II) and control group
	p value	p value
S	**0.0000**	**0.0002**
Fe	0.0843	0.9057
Br	**0.0000**	**0.0000**

10S Table. The probability p values for the t-test comparing the mean values of element concentrations in the studied group of men (measurement I and measurement II) and in the control group.

Element	studied group of men (measurement I) and control group		studied group of men (measurement II) and control group	
	p value for mean value	p value for variance	p value for mean value	p value for variance
ln P	0.1069	0.5713	**0.0010**	**0.0048**
ln S	**0.0000**	0.0937	**0.0000**	0.2304
ln Cl	0.1715	0.4568	0.1222	0.1178
ln K	0.7271	**0.0237**	0.4048	0.0837
ln Ca	**0.0463**	0.0619	0.1643	0.0923
ln Cr	**0.0418**	0.2138	0.4362	0.9880
ln Fe	**0.0192**	0.8819	0.3894	0.0539
ln Cu	**0.0431**	0.5261	**0.0008**	0.8436
ln Zn	0.1760	**0.0385**	0.2623	0.0546
ln Se	**0.0058**	**0.0034**	**0.0248**	**0.0072**
ln Br	**0.0000**	**0.0002**	**0.0000**	**0.0022**

11S Table. The probability p values for the nonparametric Mann-Whitney test comparing the element concentration distributions in the studied group of men (measurement I and measurement II) and in the control group.

Element	studied group of men (measurement I) and control group	studied group of men (measurement II) and control group
	p value	p value
P	0.0786	**0.0101**
S	**0.0001**	**0.0000**
K	0.9372	0.3314
Zn	0.1346	0.3314
Se	**0.0063**	**0.0294**
Br	**0.0000**	**0.0000**

12S Table. Correlation between element concentrations (after the logarithmic transformation) in the control group. Pearson's correlation coefficients statistically different than zero are marked in bold.

Logarithm of element content	Pearson correlation coefficient Control group										
	ln P	ln S	ln Cl	ln K	ln Ca	ln Cr	ln Fe	ln Cu	ln Zn	ln Se	ln Br
ln P	-	0.1580	0.0901	0.1740	**0.3742**	-0.2321	0.0669	**0.4614**	-0.0758	-0.0519	0.0633
ln S	0.1580	-	0.2168	0.1649	**0.7204**	**-0.5148**	**0.3361**	0.1076	**0.4052**	**0.3638**	0.1692
ln Cl	0.0901	0.2168	-	**0.3759**	**0.3886**	-0.1370	-0.1439	-0.0198	0.0849	0.0135	0.1079
ln K	0.1740	0.1649	**0.3759**	-	0.2771	-0.1222	0.0751	0.1978	**0.4766**	0.1685	**0.3137**
ln Ca	**0.3742**	**0.7204**	**0.3886**	0.2771	-	**-0.4049**	0.2439	**0.2806**	**0.4329**	**0.4440**	0.0963
ln Cr	-0,2321	**-0.5148**	-0.1370	-0.1222	**-0.4049**	-	-0.0789	-0.0596	-0.1163	-0.0601	0.1418
ln Fe	0.0669	**0.3361**	-0.1439	0.0751	0.2439	-0.0789	-	0.0314	**0.3943**	0.1073	0.1355
ln Cu	**0.4614**	0.1076	-0.0198	0.1978	**0.2806**	-0.0596	0.0314	-	0.0372	0.2254	0.1280
ln Zn	-0.0758	**0.4052**	0.0849	**0.4766**	**0.4329**	-0.1163	**0.3943**	0.0372	-	**0.4923**	**0.3413**
ln Se	-0.0519	**0.3638**	0.0135	0.1685	**0.4440**	-0.0601	0.1073	0.2254	**0.4923**	-	**0.4289**
ln Br	0.0633	0.1692	0.1079	**0.3137**	0.0963	0.1418	0.1355	0.1280	**0.3413**	**0.4289**	-

13S Table. Correlation between element concentrations (after logarithmic transformation) in studied group (measurement I). Pearson correlation coefficients statistically different than zero are marked in bold.

Logarithm of element content measurement I	Pearson correlation coefficient Studied group (measurement I)										
	ln P	ln S	ln Cl	ln K	ln Ca	ln Cr	ln Fe	ln Cu	ln Zn	ln Se	ln Br
ln P	-	-0.1127	0.1841	0.1626	**0.2890**	**-0.3001**	0.1916	0.2706	0.2453	0.2821	-0.2027
ln S	-0.1127	-	-0.1351	0.2297	**0.4405**	-0.2704	-0.1728	0.2194	0.2674	0.2215	-0.1951
ln Cl	0.1841	-0.1351	-	0.1283	0.2091	-0.0159	0.2209	-0.1108	0.0618	0.1008	**0.4126**
ln K	0.1626	0.2297	0.1283	-	**0.3775**	-0.2096	-0.0317	0.0513	0.1833	0.0961	0.0009
ln Ca	**0.2890**	**0.4405**	0.2091	**0.3775**	-	**-0.4596**	-0.1971	**0.4837**	0.1874	**0.5012**	-0.0953
ln Cr	**-0.3001**	-0.2704	-0.0159	-0.2096	**-0.4596**	-	0.0710	-0.2435	-0.2097	-0.2587	0.0134
ln Fe	0.1916	-0.1728	0.2209	-0.0317	-0.1971	0.0710	-	-0.2480	0.2764	-0.1043	**0.3009**
ln Cu	0.2706	0.2194	-0.1108	0.0513	**0.4837**	-0.2435	-0.2480	-	**0.3531**	**0.6520**	-0.1235
ln Zn	0.2453	0.2674	0.0618	0.1833	0.1874	-0.2097	0.2764	**0.3531**	-	**0.4392**	-0.0302
ln Se	0.2821	0.2215	0.1008	0.0961	**0.5012**	-0.2587	-0.1043	**0.6520**	**0.4392**	-	0.0944
ln Br	-0.2027	-0.1951	**0.4126**	0.0009	-0.0953	0.0134	**0.3009**	-0.1235	-0.0302	0.0944	-

14S Table. Correlation between element concentrations (after logarithmic transformation) in studied group (measurement II). Pearson correlation coefficients statistically different than zero are marked in bold.

Logarithm of element content measurement II	Pearson correlation coefficient Studied group (measurement II)										
	ln P	ln S	ln Cl	ln K	ln Ca	ln Cr	ln Fe	ln Cu	ln Zn	ln Se	ln Br
ln P	-	-0.2211	-0.0126	0.0842	0.2562	0.0781	0.0542	**0.3503**	-0.0562	0.1440	0.0621
ln S	-0.2211	-	0.1364	0.2171	**0.4162**	**-0.3342**	-0.2041	0.1513	**0.3000**	0.2033	-0.0954
ln Cl	-0.0126	0.1364	-	**0.2871**	**0.6173**	-0.1578	-0.0285	-0.1839	-0.0041	-0.1294	**0.3024**
ln K	0.0842	0.2171	**0.2871**	-	**0.5587**	-0.2720	0.0876	0.0667	0.1570	0.0502	0.2320
ln Ca	0.2562	**0.4162**	**0.6173**	**0.5587**	-	**-0.2881**	0.0029	0.1821	0.0478	0.1577	0.1908
ln Cr	0.0781	**-0.3342**	-0.1578	-0.2720	**-0.2881**	-	0.2831	0.0141	0.1792	0.0357	0.1681
ln Fe	0.0542	-0.041	-0.0285	0.0876	0.0029	0.2831	-	-0.0362	0.1179	-0.0677	0.0636
ln Cu	**0.3503**	0.1513	-0.1839	0.0667	0.1821	0.0141	-0.0362	-	**0.3292**	**0.3575**	0.0894
ln Zn	-0.0562	**0.3000**	-0.0041	0.1570	0.0478	0.1792	0.1179	**0.3292**	-	**0.3530**	0.2467
ln Se	0.1440	0.2033	-0.1294	0.0502	0.1577	0.0357	-0.0677	**0.3575**	**0.3530**	-	0.2297
ln Br	0.0621	-0.0954	**0.3024**	0.2320	0.1908	0.1681	0.0636	0.0894	0.2467	0.2297	-

15S Table. Correlation between element concentrations (after logarithmic transformation) in the studied group obtained in measurement I and measurement II). Pearson correlation coefficients statistically different than zero are marked in bold.

Logarithm of element content	Pearson correlation coefficient Studied group (measurement I and II)
ln P	**0.5588**
ln S	**0.8388**
ln Cl	**0.3118**
ln K	**0.3789**
ln Ca	0.0778
ln Cr	0.1977
ln Fe	0.1814
ln Cu	**0.4191**
ln Zn	**0.5702**
ln Se	**0.6024**
ln Br	**0.7170**
