# Supplementary material for: Study of element concentrations in blood serum of patients receiving parenteral nutrition using total reflection X-ray fluorescence analysis
Source: PLoS One. 2026 Feb 2;21(2):e0341090. doi: 10.1371/journal.pone.0341090 (PMC12863542; doi:10.1371/journal.pone.0341090)
Supplement: Attachment — Submitted filename: Peer Review Report.pdf [file pone.0341090.s002.pdf]

Peer Review Report

Summary & Overall Impression

Thank you for the opportunity to review this manuscript. It addresses an important and underexplored area of clinical trace-element monitoring and demonstrates the application of total reflection X-ray fluorescence (TXRF) in a novel clinical context.

The manuscript presents an original and technically rigorous study quantifying macro- and microelements in serum samples from patients receiving parenteral nutrition (PN). Measurements were performed on the first and seventh days of PN, compared with a control group, and further analyzed for gender-related differences and inter-element correlations.

The work is methodologically sound and contributes valuable data to the fields of clinical nutrition and elemental analysis. It demonstrates meticulous analytical execution and appropriate statistical treatment. The primary revisions required involve clarifying methodological details (e.g., sample timing, replicates, and calibration procedures), improving data presentation for clarity, and expanding the biological and clinical interpretation of the results.

Overall, the data appear robust, the analytical methodology is well validated, and the conclusions are supported by the presented evidence.

Overall recommendation: Minor Revision – Accept after clarification and editorial improvements.

Comments & Suggestions

1. Sampling Design and Study Cohort

Please expand the description of sampling and participant selection to enhance transparency and reproducibility:

- Clarify whether *Measurement I* represents pre-PN baseline samples or samples collected within 24 hours of PN initiation, and whether *Measurement II* occurred exactly seven days after initiation.
- Indicate if samples were collected under fasting conditions, at a standardized time of day, and under identical conditions for all participants.
- Describe how control subjects were recruited and matched to patients (age, sex, and clinical status).
- Specify whether measurements were based on technical or biological replicates and whether reported values represent single determinations or averaged replicates.
- Include information on sample handling and storage (temperature, duration, number of freeze–thaw cycles) and describe how potential hemolysis was assessed or mitigated.

2. Analytical Methodology and TXRF Performance

To strengthen reproducibility and confidence in the analytical results, please elaborate on key instrumental details:

- Identify any internal standard used for TXRF quantification, its concentration, and the calibration procedure.
- Indicate how frequently instrument calibration was verified and whether blank or drift-control measurements were performed between sample runs.
- Describe how data points near the detection limit were treated statistically (excluded, substituted, or handled using censored-data approaches).
- Clarify whether logarithmic transformations were applied prior to statistical testing and whether mean values were back-transformed for presentation.
- If replicate analyses were conducted, specify whether these were technical or biological replicates and report the typical within-sample variability observed.

3. Data Presentation and Statistical Reporting

The Results section is comprehensive, but a more concise summary would improve readability and highlight the main findings:

- Provide a clearer overview of which elements exhibited the most notable changes between *Measurements I* and *II* (for example, Cu and Se).
- Some elements show considerable variability (e.g., Fe, Zn, Cr). Please clarify whether this variability arises from biological diversity or analytical uncertainty, and describe any treatment of outliers.
- State whether paired statistical tests were applied for within-subject comparisons or whether each group was analyzed independently.

4. Biological and Clinical Interpretation

The Discussion would be strengthened by a more detailed interpretation of observed elemental trends:

- Elaborate on possible physiological or biochemical mechanisms that might explain increased Cu and Se concentrations after PN and the relative stability of Zn and Fe.
- Discuss whether these changes are clinically significant in relation to established reference ranges or deficiency/toxicity thresholds.
- For gender-based comparisons, comment on whether the sample size provided adequate statistical power and whether hormonal or metabolic factors might contribute to observed differences.

5. Correlations and Element Relationships

Interpretation of inter-element correlations would benefit from additional context:

- Some correlations (e.g., Ca–S positive; Cr–Ca negative) appear consistent across measurements. Could these patterns reflect shared metabolic pathways or PN formulation effects?
- Given the large number of correlations tested, please discuss whether weaker correlations ($r \approx 0.2$–0.3) should be considered biologically meaningful or could represent statistical chance.

6. Study Limitations and Future Work

Please broaden the Limitations section to acknowledge:

- The modest cohort size and short (one-week) observation period.
- Potential variability in PN composition and in patients' clinical status.
- Consider suggesting future directions such as larger or longer studies, inclusion of additional trace elements, or correlation of elemental shifts with clinical recovery parameters.

7. Clinical and Translational Relevance

The work would benefit from a brief discussion of potential clinical applications:

- Could multi-element TXRF profiling be implemented for PN monitoring or formulation adjustments in hospital settings?
- Based on your findings, which elements might be most informative for routine monitoring given their variability and clinical significance?

8. Presentation, Style, and Minor Edits

For clarity and polish:

- Integrate brief interpretive remarks into the Results section (e.g., "Cu increased by 18 %, consistent with PN supplementation") to guide readers through key findings.
- Correct minor typographical errors and ensure consistent terminology and formatting throughout:
  "Univeristy" → "University"; *"wass" → "was"*; *"ration" → "ratio"*
- Consider concluding the Discussion with a concise paragraph summarizing the principal findings, their biological implications, and the broader significance for clinical nutrition.

Overall Assessment

This manuscript is methodologically sound, original, and ethically compliant. The TXRF methodology is clearly validated, and the data support the authors' conclusions. The suggested revisions are primarily clarifications and interpretive enhancements rather than experimental additions. Addressing them will significantly improve the clarity, transparency, and clinical relevance of the work.

Recommendation: *Minor Revision – Accept after addressing methodological and interpretive comments.*
