# Supplementary material for: Study of element concentrations in blood serum of patients receiving parenteral nutrition using total reflection X-ray fluorescence analysis
Source: PLoS One. 2026 Feb 2;21(2):e0341090. doi: 10.1371/journal.pone.0341090 (PMC12863542; doi:10.1371/journal.pone.0341090)
Supplement: Attachment — Submitted filename: Response to Reviewers.docx [file pone.0341090.s004.docx]

Kielce, 17.11.2025

Satish Rojekar, Ph.D.

Academic Editor

PLOS ONE

Journal: PLOS ONE

Manuscript Number: PONE-D-25-39141

Title: Study of element concentrations in blood serum of patients receiving parenteral nutrition using total reflection X-ray fluorescence analysis

Authors: Monika Pierzak-Stępień, Aldona Kubala-Kukuś, Dariusz Banaś, Ilona Stabrawa, Jolanta Wudarczyk-Moćko, Karol Szary, Monika Biernacka, Milena Piotrowska, Dariusz Pasieka, Natalia Wojtaś, Andrzej Dąbrowski, Stanisław Głuszek

Authors would like to thank the Editor and Reviewers for their accurate comments and suggestions which taking into account in the manuscript have essentially improved the quality of the paper.

Note: The manuscript lines quoted in response to the reviewers refer to the revised manuscript without track changes.

**Journal Requirements**:

Question 1. Please ensure that your manuscript meets PLOS ONE's style requirements, including those for file naming. The PLOS ONE style templates can be found at

<https://journals.plos.org/plosone/s/file?id=wjVg/PLOSOne_formatting_sample_main_body.pdf> and

<https://journals.plos.org/plosone/s/file?id=ba62/PLOSOne_formatting_sample_title_authors_affiliations.pdf>

Authors' response: Thank to the editor for this note. The manuscript was checked in context of PLOS ONE’s style reguirements.

Question 2. Thank you for stating the following financial disclosure:

“The functioning of the facility is supported by Polish Ministry of Education and Science (project 28/489259/SPUB/SP/2021).

This work was co-financed by the Minister of Science (Poland) under the "Regional Excellence Initiative" program (project no.: RID/SP/0015/2024/01).”

Please state what role the funders took in the study.  If the funders had no role, please state: "The funders had no role in study design, data collection and analysis, decision to publish, or preparation of the manuscript."

Authors' response: Thank to the editor for this note. The funders had no role in the study. We state: "The funders had no role in study design, data collection and analysis, decision to publish, or preparation of the manuscript." This information was included in the Cover letter.

Question 3. Thank you for stating the following in the Acknowledgments Section of your manuscript:

“The functioning of the facility is supported by Polish Ministry of Education and Science (project 28/489259/SPUB/SP/2021).

This work was co-financed by the Minister of Science (Poland) under the "Regional Excellence Initiative" program (project no.: RID/SP/0015/2024/01).”

We note that you have provided additional information within the Acknowledgements Section that is not currently declared in your Funding Statement. Please note that funding information should not appear in the Acknowledgments section or other areas of your manuscript. We will only publish funding information present in the Funding Statement section of the online submission form.

Please remove any funding-related text from the manuscript and let us know how you would like to update your Funding Statement. Currently, your Funding Statement reads as follows:

“The functioning of the facility is supported by Polish Ministry of Education and Science (project 28/489259/SPUB/SP/2021).

This work was co-financed by the Minister of Science (Poland) under the "Regional Excellence Initiative" program (project no.: RID/SP/0015/2024/01).”

Please include your amended statements within your cover letter; we will change the online submission form on your behalf.

Authors' response: Thank to the editor for this note. We removed the funding information from Acknowledgments section of our manuscript. In the Cover letter we specified the information we wanted to include in the Funding Statement.

Question 4. If the reviewer comments include a recommendation to cite specific previously published works, please review and evaluate these publications to determine whether they are relevant and should be cited. There is no requirement to cite these works unless the editor has indicated otherwise. 
Authors' response: Thank to the editor for this note. The reviewer comments did not include a recommendation to cite specific previously published works.

Question 5. To ensure your figures meet our technical requirements, please review our figure guidelines: https://journals.plos.org/plosone/s/figures

You may also use PLOS’s free figure tool, NAAS, to help you prepare publication quality figures: https://journals.plos.org/plosone/s/figures#loc-tools-for-figure-preparation.

NAAS will assess whether your figures meet our technical requirements by comparing each figure against our figure specifications.

Authors' response: Thank to the editor for this note. The figures were checked and corrected.

**Reviewers' comments to the Author**:

**Reviewer #1**

Question 6. It would be valuable to briefly mention potential directions for future research. For example, could the study be expanded to other patient populations, or might it be used to refine more personalized parenteral nutrition protocols?

Authors' response: Thank to the reviewer for this note. Information was added in the Conclusions.

In fact, the multi-element TXRF profiling of human serum samples is used by use in routine, daily, analysis of serum of Holycross Cancer Center patients (Kielce, Poland). We typically analyze the serum of individuals with Hodgkin's lymphoma for copper content and the serum of patients treated with selenium preparations.

Question 7. A brief mention of any limitations or potential sources of bias would be beneficial. Discussing aspects such as sample size, study duration, or patient demographics could help contextualize the results and provide a balanced perspective.

Authors' response: Thank to the reviewer for this note. In the conclusions the following sentence was added: ,,In the future, it is planned to increase the number of patients in the study and control groups and to improve the gender balance.” (line 756).

**Reviewer #2**

Question 8. Abstract should more clearly state the key findings and clinical significance rather than extensive methodological details.

Authors' response: Thank to the reviewer for this note. Abstract was corrected.

Question 9. In the introduction, background on parenteral nutrition is comprehensive but it’s too lengthy. It could be revised to highlight the key research gap.

Authors' response: Thank to the reviewer for this note. Introduction was corrected.

Question 10. The study group is relatively small (n=48), and gender/age distribution between study and control groups is not perfectly balanced. This should be discussed as a limitation.

Authors' response: Thank to the reviewer for this note. In the conclusions the following sentence was added: ,,In the future, it is planned to increase the number of patients in the study and control groups and to improve the gender balance.” (line 756).

Question 11. The method is well described, but reproducibility and quality control data should be more clearly summarized. For instance, detection limits and uncertainty levels are scattered in the text. These should be presented in a concise table.

Authors' response: Thank to the reviewer for this note. We prepared table with discussed information (Table 1).

Question 12. The clinical implications of the findings are underdeveloped. For example, how might the observed Se and Cu differences affect nutritional protocols or patient monitoring? Are these differences clinically significant, or only statistically significant?

Authors' response: Thank to the reviewer for this note. In fact, the multi-element TXRF profiling of human serum samples is used by use in routine, daily, analysis of serum of Holycross Cancer Center patients (Kielce, Poland). We typically analyze the serum of individuals with Hodgkin's lymphoma for copper content and the serum of patients treated with selenium preparations. Therefore, implementation of PN monitoring, especially in the context of formulation adjustments in hospital settings, can be done. In the section Conclusions we added the following information: ,,In conclusion, it can be stated that the use of comprehensive information obtained from the simultaneous analysis of the element contents in serum using the TXRF technique and from statistical analysis, enables assessing the health status of the patients receiving parenteral nutrition and planning more personalized parenteral nutrition protocols. In the future, it is planned to increase the number of patients in the study and control groups and to improve the gender balance.”

Question 13. Results are heavily tabulated. Adding graphical visualizations (boxplots, forest plots, heatmaps) would improve clarity.

Authors' response: Thank to the reviewer for this note. The obtained results are presented graphically in new Figure 1.

Question 14. Ensure consistent use of abbreviations (e.g., CRP, NRS, BMI).

Authors' response: Thank to the reviewer for this note. Abbreviations are now explained when they first appear in the text.

**Reviewer #3**

Sampling Design and Study Cohort

Question 15. Clarify whether Measurement I represents pre-PN baseline samples or samples collected within 24 hours of PN initiation, and whether Measurement II occurred exactly seven days after initiation.

Author’s response: Thank to the reviewer for this note. Measurement I represents pre-PN baseline samples. Measurement II occurred exactly seven days after initiation. Information was included in the paper.

Question 16. Indicate if samples were collected under fasting conditions, at a standardized time of day, and under identical conditions for all participants.

Author’s response: Thank to the reviewer for this note. In the study we included the patients who received intravenous (parenteral) nutrition as their sole source of nutrients and non-nutritive components. Nutritional status and serum trace element concentrations were assessed twice, before the nutritional therapy and on the seventh day. Blood samples were collected in the morning, before the feeding bag was connected, in the surgical room of the Department of Surgery. Each patient underwent daily clinical monitoring during nutritional therapy, including fluid balance, temperature and body weight measurements, and monitoring of the central and/or peripheral venous catheter. This information was also included in the manuscript (line 182, line 195).

Question 17. Describe how control subjects were recruited and matched to patients (age, sex, and clinical status).

Author’s response: Thank to the reviewer for this note. In the paper we included the following information: ,,In the study, for comparison purposes, also the group of patients not receiving parenteral nutrition, was included. This control group consisted of 50 patients (35 women and 15 men) scheduled for planned gallbladder removal surgery (cholecystectomy), aged 30-80. Criteria for including patients within the control group were: age (from 30 to 80 years), patients admitted for planned cholecystectomy, without symptoms of cholecystitis, normal nutritional status and obtaining informed consent from the patient to participate in the study. Among women, the average age (in years) was 53 and median was 50. The youngest woman was 30, while the oldest was 80. Among men, the average age (in years) was 54 and median was 60. The youngest man was 30, while the oldest was 80 [8]. For the patients in the control group, all anthropometric, biochemical and immunological parameters and elemental analysis of the serum were measured once, before cholecystectomy.” (lines 183-194).

Question 18. Specify whether measurements were based on technical or biological replicates and whether reported values represent single determinations or averaged replicates.

Author’s response: Thank to the reviewer for this note. In TXRF serum sample analysis, for each sample the duplicate measurements were performed. Next, the average value was reported. The observed variability between measurements depends on the given element and its concentration in the sample and is on the level from about 0.1% to 20%. This information was now included in the manuscript.

Question 19. Include information on sample handling and storage (temperature, duration, number of freeze–thaw cycles) and describe how potential hemolysis was assessed or mitigated.

Author’s response: Thank to the reviewer for this note. The information was included in the text (line 211).

Analytical Methodology and TXRF Performance

Question 20. Identify any internal standard used for TXRF quantification, its concentration, and the calibration procedure.

Authors' response: Serum samples of the patients from the studied and control groups were prepared according to the following procedure: 0.8 mL of a sample was mixed with 0.05 mL of Ga (100 μg/g) used as an internal standard (line 236 in the paper). The qualitative analysis of the X-ray spectrum and the quantitative analysis of the sample elemental composition were done using spectrometer software (SPECTRA 7) based on the spectrometer calibration (line 215).

Question 21. Indicate how frequently instrument calibration was verified and whether blank or drift-control measurements were performed between sample runs.

Author’s response: Thank to the reviewer very much for this note. Now we included in the text: ,,The instrument calibration is performed each day at the beginning of the measurements. The TXRF analysis validation was performed by investigating reference control serum solution at the beginning of the studied sample measurement.” (line 215)

Question 22. Describe how data points near the detection limit were treated statistically (excluded, substituted, or handled using censored-data approaches).

Author’s response: Thank to the reviewer for this note. The data points near the detection limit were handled using censored-data statistical approach. This information was included in the manuscript (line 271).

Question 23. Clarify whether logarithmic transformations were applied prior to statistical testing and whether mean values were back-transformed for presentation.

Author’s response: Thank to the reviewer for this note. The parameters of descriptive statistics were calculated based on raw data. This information was included in the manuscript (lines 286 and 307).

The logarithmic transformations were applied for statistical testing.

Question 24. If replicate analyses were conducted, specify whether these were technical or biological replicates and report the typical within-sample variability observed.

Author’s response: Thank to the reviewer for this note. In TXRF serum sample analysis, for each sample the duplicate measurements were performed. The observed variability depends on the given element and its concentration in the sample and is on the level from about 0.1% to 20%. This information was now included in the manuscript.

Data Presentation and Statistical Reporting

Question 25. Provide a clearer overview of which elements exhibited the most notable changes between Measurements I and II (for example, Cu and Se).

Author’s response: Thank to the reviewer for this note. Increased Cu and Se concentrations after parenteral nutrition can be interpreted in the context of their specific metabolic roles, transport proteins and supplementation practices used during parenteral nutrition. Copper is added routinely to parenteral mixtures in standardized trace-element formulations. Supplementation, together with improved hepatic protein synthesis after initiation of PN, enhances the production of ceruloplasmin, which is the main plasma Cu-binding protein. Ceruloplasmin concentration responds quickly to changes in nutritional status and acute-phase reactions. A rise in ceruloplasmin increases circulating Cu, even when total body copper stores change modestly. In addition, reduced intestinal losses due to minimal enteral stimulation and improved overall metabolic balance during PN support better retention of supplemented Cu. Selenium was administered as part of standard PN supplementation. Its plasma concentration reflects selenite availability and rapid incorporation into selenoproteins such as glutathione peroxidase. PN provides controlled Se input independent of gastrointestinal absorption efficiency, which is often impaired in critically ill or malnourished patients. Restoration of redox balance and improved hepatic synthesis after several days of PN facilitate incorporation of Se into plasma selenoproteins, leading to measurable increases in serum Se concentration.

Summary: the increase in Cu and Se reflects direct supplementation, efficient transport protein upregulation and rapid incorporation into functional proteins.

This information, in shorter version, was included in the paper (line 482).

Question 26. Some elements show considerable variability (e.g., Fe, Zn, Cr). Please clarify whether this variability arises from biological diversity or analytical uncertainty, and describe any treatment of outliers.

Author’s response: Thank to the reviewer for this note. This variability arises from biological diversity not from analytical uncertainty. We have extensive experience analyzing the distributions of elemental content in various human biological samples (serum, urine, hair, tumor tissue, placenta, fetal membranes). These distributions are always asymmetric, with large standard deviations, suggesting a high population dispersion of elemental content. In general, we have shown (paper *) that elemental content distributions in such samples are described by log-stable distributions, with the lognormal distribution being a specific example of a log-stable distribution. A characteristic feature of these distributions is the occurrence of extremely small or extremely large elemental content values (over three standard deviations, as tested for outliers), which is a natural observation. In our article, we provided informative numerical values of many descriptive statistics parameters because numerical information, not only graphical, is very useful in clinical practice, for example, for defining reference ranges for elemental content. However, for log-stable distributions, the best measures of location parameters are the median and quartiles because the presence of outliers does not change the values of these parameters. Some of this explanation has now been added to the article (line 340).

* A. Kubala-Kukuś, E. Kuternoga, J. Braziewicz, M. Pajek, Log-stable Concentration Distributions of Trace Elements in Biomedical Samples, Spectrochimica Acta B 59 (2004) 1711-1716, DOI: 10.1016/j.sab.2004.07.015

Question 27. State whether paired statistical tests were applied for within-subject comparisons or whether each group was analyzed independently.

Author’s response: The paired statistical tests were applied for comparison results of measurement I and measurement II. (Text in the manuscript: ,,Verification of the hypothesis related to the comparing the mean values of the element contents in the measurement I and measurement II of the studied group were performed using the t-test for dependent (paired) samples (STATISTICA).”, line 436).

Biological and Clinical Interpretation

Question 28. Elaborate on possible physiological or biochemical mechanisms that might explain increased Cu and Se concentrations after PN and the relative stability of Zn and Fe.

Author’s response: Thank to the reviewer for this note. Increased Cu and Se concentrations after parenteral nutrition and the relative stability of Zn and Fe can be interpreted in the context of their specific metabolic roles, transport proteins and supplementation practices used during parenteral nutrition. Copper is added routinely to parenteral mixtures in standardized trace-element formulations. Supplementation, together with improved hepatic protein synthesis after initiation of PN, enhances the production of ceruloplasmin, which is the main plasma Cu-binding protein. Ceruloplasmin concentration responds quickly to changes in nutritional status and acute-phase reactions. A rise in ceruloplasmin increases circulating Cu, even when total body copper stores change modestly. In addition, reduced intestinal losses due to minimal enteral stimulation and improved overall metabolic balance during PN support better retention of supplemented Cu. Selenium was administered as part of standard PN supplementation. Its plasma concentration reflects selenite availability and rapid incorporation into selenoproteins such as glutathione peroxidase. PN provides controlled Se input independent of gastrointestinal absorption efficiency, which is often impaired in critically ill or malnourished patients. Restoration of redox balance and improved hepatic synthesis after several days of PN facilitate incorporation of Se into plasma selenoproteins, leading to measurable increases in serum Se concentration. Unlike Cu and Se, Zn concentration remains relatively stable in serum despite supplementation. Plasma Zn is tightly homeostatically regulated and only a small fraction reflects total body stores. Redistribution of Zn to tissues (especially liver and muscles) often occurs during acute-phase response and inflammation. Albumin is the main Zn carrier; its concentration in PN patients may remain unchanged or reduced due to underlying disease, limiting visible increases in serum Zn. Serum Fe concentrations mainly reflect the dynamics of transferrin-bound Fe, not total Fe availability. PN typically does not include iron due to the risk of oxidative stress and infectious complications, so no direct Fe supplementation occurs. Moreover, inflammatory states and metabolic stress common in PN patients trigger increased hepcidin synthesis, which decreases iron release from macrophages and enterocytes. As a result, serum Fe remains stable or variable but without a consistent upward trend.

Summary: the increase in Cu and Se reflects direct supplementation, efficient transport protein upregulation and rapid incorporation into functional proteins, while Zn and Fe remain stable due to stricter homeostatic control and, in the case of Fe, absence of supplementation and inflammation-related regulation through hepcidin.

This information, in shorter version, was included in the paper (line 482).

Question 29. Discuss whether these changes are clinically significant in relation to established reference ranges or deficiency/toxicity thresholds.

Author’s response: Thank to the reviewer for this note. These changes are clinically significant, especially in the context of Se, because the effectiveness of Se supplementation and the consistency of the selenium content with the value for the control group were confirmed.

Question 30. For gender-based comparisons, comment on whether the sample size provided adequate statistical power and whether hormonal or metabolic factors might contribute to observed differences.

Author’s response: Thank to the reviewer for this note. In our research we do not observe the differences between the concentration distributions in the groups of women and men. In order to improve the statistical power of hypotheses verification we are going to increase the number of patients in the study group.

Correlations and Element Relationships

Question 31. Some correlations (e.g., Ca–S positive; Cr–Ca negative) appear consistent across measurements. Could these patterns reflect shared metabolic pathways or PN formulation effects?

Author’s response: Thank to the reviewer for this note. The recurring correlations (positive Ca–S, negative Cr–Ca) likely reflect underlying metabolic relationships, but PN formulation may also contribute. Positive Ca–S correlation: sulfur-containing amino acids (cysteine, methionine) and sulfate participate in protein-bound calcium buffering and in maintaining acid–base balance. Both Ca and S rise or fall together when plasma protein synthesis or hydration status changes. PN mixtures provide amino acids and electrolytes in fixed proportions, which may reinforce this coordinated pattern. This explains the persistence of a positive Ca–S relationship across all measurements and in the control group. Negative Cr–Ca correlation: chromium and calcium are absorbed and transported via partly competing ionic mechanisms. Chromium’s role in insulin signaling may shift distribution toward intracellular compartments, while calcium homeostasis is tightly regulated by albumin, parathyroid hormone and vitamin D. In catabolic or inflammatory conditions, Ca tends to remain relatively stable, whereas Cr exhibits wider variability. Information is included in the paper (line 611).

The reproducibility of these patterns suggests that they arise from a combination of: 1) shared physiological regulation (protein binding, transport processes), and 2)	fixed nutrient proportions in PN mixtures, which stabilize certain element relationships while allowing others (e.g., Cr) to vary more freely.

Question 32. Given the large number of correlations tested, please discuss whether weaker correlations (r ≈ 0.2–0.3) should be considered biologically meaningful or could represent statistical chance.

Author’s response: Thank to the reviewer for this question. In our opinion the correlations confirmed as the statistically significant can be considered as biologically meaningful.

Study Limitations and Future Work

Question 33. The modest cohort size and short (one-week) observation period. Potential variability in PN composition and in patients’ clinical status. Consider suggesting future directions such as larger or longer studies, inclusion of additional trace elements, or correlation of elemental shifts with clinical recovery parameters.

Authors' response: Thank to the reviewer for this note. In the conclusions the following sentence was added: ,,In the future, it is planned to increase the number of patients in the study and control groups and to improve the gender balance.” (line 756).

Clinical and Translational Relevance

Question 34. Could multi-element TXRF profiling be implemented for PN monitoring or formulation adjustments in hospital settings?

Author’s response: Thank to the reviewer for this question. In fact, the multi-element TXRF profiling of human serum samples is used by use in routine, daily, analysis of serum of Holycross Cancer Center patients (Kielce, Poland). We typically analyze the serum of individuals with Hodgkin's lymphoma for copper content and the serum of patients treated with selenium preparations. Therefore, implementation of PN monitoring, especially in the context of formulation adjustments in hospital settings, can be done.

Question 35. Based on your findings, which elements might be most informative for routine monitoring given their variability and clinical significance?

Authors' response: Thank to the reviewer for this note. In our opinion the most informative elements for routine monitoring are Se and Cu.

Presentation, Style, and Minor Edits

Question 36. Integrate brief interpretive remarks into the Results section (e.g., “Cu increased by 18 %, consistent with PN supplementation”) to guide readers through key findings.

Authors’ response: Thank to the reviewer for this note. The information was included (line 475).

Question 37. Correct minor typographical errors and ensure consistent terminology and formatting throughout: “Univeristy” → “University”; “wass” → “was”; “ration” → “ratio”

Authors' response: Thank to the reviewer for this note. It was corrected.

Question 38. Consider concluding the Discussion with a concise paragraph summarizing the principal findings, their biological implications, and the broader significance for clinical nutrition.

Authors' response: Thank to the reviewer for this note. The information was included in section Conclusions (line 712).

**Reviewer #4**

Question 39. Refine the Abstract's Focus: Streamline the abstract to highlight the main hypothesis and two or three most impactful findings. For example, focus on the differences between baseline (Meas. I) and post-PN (Meas. II) for the supplemented elements (Cr, Se) and for any critical elements found to be deficient/excessive. Do not include specific correlation coefficients (0.4-0.7) or an exhaustive list of all differing elements (P, S, Ca, Fe...).

Authors' response: Thank to the reviewer for this note. Abstract was corrected according the reviewer’s suggestions.

Question 40. Clearly State the Direction of Change: When mentioning differences in the abstract, clarify the direction. Instead of, "Comparing element concentrations for measurement I and II indicates the differences in Cu and Se," write, "Parenteral nutrition resulted in a significant increase in supplemented Se and a decrease in Cu concentrations between measurement I and II."

Authors' response: Thank to the reviewer for this note. This sentence was corrected according the reviewer’s suggestion.

Question 41. Strengthen Control Group Justification: In the Introduction or Methods, briefly state why the cholecystectomy patients were deemed an appropriate control. This bolsters the argument that their elemental levels represent a healthy baseline, despite being hospitalized for surgery.

Authors' response: Thank to the reviewer for this note. The control group consisted of individuals admitted for planned gallbladder removal without nutritional deficiencies. Patients admitted for planned cholecystectomy were intended to be representative of serum trace element concentrations, as they had normal nutritional status. Nutritional status among the control group was verified using a self-developed questionnaire, the Nutritional Risk Screening 2002 (NRS) malnutrition risk assessment form, and anthropometric measurements. Serum trace element concentrations were measured once before the surgical cholecystectomy.

Question 42. For the Methods and Results Sections Enhance Data Visualization: The presentation of descriptive statistics (Tables 1 and 2) is detailed, but the results section would be far more accessible with clear visualization, such as box-and-whisker plots or bar charts. This is essential for illustrating the distributions, outliers (especially for Fe, as indicated by the high standard deviation), and group differences (Control vs. Studied I vs. Studied II).

Authors' response: Thank to the reviewer for this note. The obtained results are presented graphically in new Figure 1.

Question 43. Address High Variability (Fe): The extremely high variation coefficients for Iron (Fe) in the studied group (138% and 218%) are concerning. This suggests the presence of significant outliers or heterogeneous patient conditions (e.g., patients with severe anemia or recent blood transfusions). The authors must address the likely source of this high variability and justify whether the outliers were included or excluded and why.

Author’s response: Thank to the reviewer for this note. This variability arises from biological diversity not from analytical uncertainty. We have extensive experience analyzing the distributions of elemental content in various human biological samples (serum, urine, hair, tumor tissue, placenta, fetal membranes). These distributions are always asymmetric, with large standard deviations, suggesting a high population dispersion of elemental content. In general, we have shown (paper *) that elemental content distributions in such samples are described by log-stable distributions, with the lognormal distribution being a specific example of a log-stable distribution. A characteristic feature of these distributions is the occurrence of extremely small or extremely large elemental content values (over three standard deviations, as tested for outliers), which is a natural observation. In our article, we provided informative numerical values of many descriptive statistics parameters because numerical information, not only graphical, is very useful in clinical practice, for example, for defining reference ranges for elemental content. However, for log-stable distributions, the best measures of location parameters are the median and quartiles because the presence of outliers does not change the values of these parameters. Some of this explanation has now been added to the article (line 341).

* A. Kubala-Kukuś, E. Kuternoga, J. Braziewicz, M. Pajek, Log-stable Concentration Distributions of Trace Elements in Biomedical Samples, Spectrochimica Acta B 59 (2004) 1711-1716, DOI: 10.1016/j.sab.2004.07.015

Question 44. Explicitly Link Elemental Changes to PN Content: Given the focus on PN, the results discussion should directly compare the measured changes to the composition of the administered PN mixture. For instance, if Se increased, confirm that the supplementation amount was appropriate for the observed change. If Cu changed, discuss if this was an intended or unintended effect of the total nutritional mixture.

Author’s response: Thank to the reviewer for this note. Increased Cu and Se concentrations after parenteral nutrition can be interpreted in the context of their specific metabolic roles, transport proteins and supplementation practices used during parenteral nutrition. Copper is added routinely to parenteral mixtures in standardized trace-element formulations. Supplementation, together with improved hepatic protein synthesis after initiation of PN, enhances the production of ceruloplasmin, which is the main plasma Cu-binding protein. Ceruloplasmin concentration responds quickly to changes in nutritional status and acute-phase reactions. A rise in ceruloplasmin increases circulating Cu, even when total body copper stores change modestly. In addition, reduced intestinal losses due to minimal enteral stimulation and improved overall metabolic balance during PN support better retention of supplemented Cu. Selenium was administered as part of standard PN supplementation. Its plasma concentration reflects selenite availability and rapid incorporation into selenoproteins such as glutathione peroxidase. PN provides controlled Se input independent of gastrointestinal absorption efficiency, which is often impaired in critically ill or malnourished patients. Restoration of redox balance and improved hepatic synthesis after several days of PN facilitate incorporation of Se into plasma selenoproteins, leading to measurable increases in serum Se concentration.

Summary: the increase in Cu and Se reflects direct supplementation, efficient transport protein upregulation and rapid incorporation into functional proteins. This information, in shorter version, was included in the paper (line 482).

Question 45. Clarity on Gender Comparison: The text mentions a difference in Sulfur (S) for the control group and Selenium (Se) for the studied group (measurement II) based on gender. This finding should be integrated more fully into the discussion, as it suggests the potential need for gender-specific PN adjustments or baseline references.

Author’s response: Thank to the reviewer for this note. This comment was included in the manuscript (line 428).
